# The catalytic ozonation of diazinon using nano-MgO@CNT@Gr as a new heterogenous catalyst: the optimization of effective factors by response surface methodology†

**DOI:** 10.1039/c9ra10095d

**Published:** 2020-02-21

**Authors:** Ghorban Asgari, Abdolmotaleb Seidmohammadi, Ali Esrafili, Javad Faradmal, Mohammad Noori Sepehr, Maghsoud Jafarinia

**Affiliations:** Social Determinants of Health Research Center (SDHRC), Hamadan University of Medical Sciences Hamadan Iran; Department of Environmental Health Engineering, School of Public Health, Hamadan University of Medical Sciences Hamadan Iran jafariniam@gmail.com m.jafarinia@abzums.ac.ir; Research Center for Environmental Health Technology, Iran University of Medical Sciences Tehran Iran; Department of Environmental Health Engineering, School of Public Health, Iran University of Medical Sciences Tehran Iran; Modeling of Noncommunicable Diseases Research Center, Hamadan University of Medical Sciences Hamadan Iran; Department of Biostatistics and Epidemiology, School of Public Health, Hamadan University of Medical Sciences Hamadan Iran; Research Center for Health, Safety and Environment, Alborz University of Medical Sciences Karaj Iran; Department of Environmental Health Engineering, School of Public Health, Alborz University of Medical Sciences Karaj Iran

## Abstract

In this research, the degradation of the insecticide diazinon was studied using a new hybrid catalyst consisting of magnesium oxide nanoparticles (nano-MgO), carbon nanotubes (CNTs), and graphite (Gr), nano-MgO@CNT@Gr, under various experimental conditions. This study shows the optimization of the nano-MgO@CNT@Gr/O_3_ process for diazinon degradation in aqueous solutions. Box–Behnken experimental design (BBD) and response surface methodology (RSM) were used to assess and optimize the solo effects and interactions of four variables, pH, catalyst loading, reaction time, and initial diazinon concentration, during the nano-MgO@CNT@Gr/O_3_ process. Analysis of regression revealed an adequate fit of the experimental results with a quadratic model, with *R*^2^ > 0.91. Following the collection of analysis of variance (ANOVA) results, pH, catalyst loading, and reaction time were seen to have significant positive effects, whereas the concentration of diazinon had a considerable negative impact on diazinon removal *via* catalytic ozonation. The four variables for maximum diazinon removal were found to be optimum (82.43%) at the following levels: reaction time, 15 min; pH, 10; catalyst dosage, 1.5 g L^−1^; and diazinon concentration, 10 mg L^−1^. The degradation of diazinon gave six kinds of by-products. The mechanism of diazinon decomposition was considered on the basis of the identified by-products. According to these results, the nano-MgO@CNT@Gr/O_3_ process could be an applicable technique for the treatment of diazinon-containing wastewater.

## Abstract 2

In this research, the degradation of the insecticide diazinon was studied using a new hybrid catalyst consisting of magnesium oxide nanoparticles (nano-MgO), carbon nanotubes (CNTs), and graphite (Gr), nano-MgO@CNT@Gr, under various experimental conditions.

## Graphical abstract

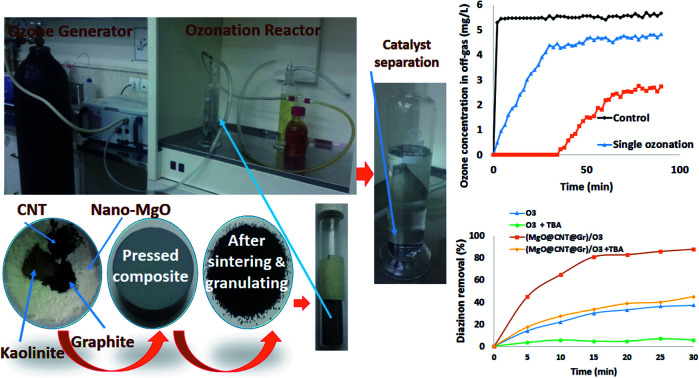

## 1. Introduction

In recent years, the release of insecticides into the environment has become a major concern.^1^ These toxic compounds have negative effects on the reproductive and central nervous systems, and they also have carcinogenic effects.^2^ Diazinon ((CH_3_)_2_CHC_4_N_2_H(CH_3_)OPS(OC_2_H_5_)_2_) is a non-selective organophosphorus insecticide. It is widely used on lettuce, turf, citrus fruits, almonds, cotton, alfalfa, and other crops and fruits.^3^ The World Health Organization (WHO) has classified diazinon as a “moderately hazardous” class II pesticide.^4,5^ The toxicity dose associated with diazinon to aquatic organisms and its fetal dose for human is 350 ng L^−1^ and 90–444 mg kg^−1^ respectively.^6^ Diazinon is a non-polar and relatively water-soluble substance, and it shows soil resistance.^7^ Hence, its presence in surface water and groundwater is worrying. Also, conventional systems are not able to remove this insecticide from water sources.^8^ The vapor pressure of diazinon is 1.4 × 10^−4^ mm Hg at 20 °C and its Henry's law constant is 1.4 × 10^−6^ atm m^3^ mol^−1^, demonstrating that it will not volatilize simply from soil and water resources.^6,7^ Advanced oxidation processes (AOPs) have been widely used for organic pollutant removal using more reactive radicals, mainly free hydroxyl radicals.^9^ AOPs can involve a combination of chemicals and UV radiation or chemicals and ozone; for example, O_3_/UV, H_2_O_2_/Fe^2+^ (Fenton's reagent), O_3_/H_2_O_2_, H_2_O_2_/UV, O_3_/H_2_O_2_/UV, and catalytic ozonation systems all exist.^10^ The combination of ozone with an active catalyst in a catalytic ozonation process (COP) increases the capability for mineralization, through converting O_3_ into very reactive hydroxyl radicals (˙OH), and increases O_3_ consumption, through enhancing the solubility of gaseous ozone.^11,12^ Catalytic ozonation has attracted researchers due to its low cost, ease of operation, increased ozone solubility, and capability for catalyst separation from the environment and its subsequent reuse.^13^ Catalytic ozonation is an acceptable technique for promoting ozonation, which is typically done in two ways. Firstly, the production of ˙OH is facilitated.^14^ Hydroxyl radicals, non-selective radicals with an excellent oxidation potential (2.80 eV) derived from ozone decomposition, have a stronger oxidizing capacity than ozone (2.07 eV) and so have greater efficiency than ozone for the degradation of recalcitrant compounds.^14,15^ Also, O_3_ directly reacts with the intermediates derived from organic materials in catalytic reactions, therefore demonstrating higher selectivity and removal efficiency towards organic pollutants.^9,14^ Catalytic ozonation can be separated into two categories: homogeneous and heterogeneous.^10^ Transition metals have been used in homogeneous catalytic ozonation processes.^16^ Many synthesized catalysts, such as metal oxides,^14,17,18^ metal oxides coated on various supports,^14,19,20^ and porous materials,^16,19,21^ have been used in heterogeneous catalytic ozonation processes. Heterogeneous catalytic ozonation is considered to encourage advanced oxidation processes in water and wastewater treatment.^21,22^ Heterogeneous catalytic ozonation shows better performance, presenting high oxidation efficiency, low O_3_ consumption, and a high reaction rate compared with single ozonation.^19,23^

Thus, herein, a new hybrid catalyst was synthesized *via* the combination of three materials in three steps: mixing, sintering, and granulation. In this work, nano-MgO, which was previously found to be effective in the degradation of refractory organic pollutants^19,20,24,25^ due to its high activity,^26,27^ chemical stability,^25,26^ and environmental friendliness,^19,28,29^ was selected as the main part of the new catalyst. Graphite (Gr) was used as a catalyst support and provided catalyst reinforcement and porosity.^28,30,31^ Carbon nanotubes were used as a support, aiding the uniform distribution of active compounds, the creation of highly active sites,^21,32^ and the enhancement of the mechanical properties of the composite.^21,33,34^ All components have been used previously in catalytic ozonation.^30,35,36^ Kaolinite was added to the mixture to form bonds between the catalyst components.^37,38^

The purpose of this study is to probe the catalytic activity of nano-MgO@CNT@Gr as a catalyst for the ozonation of diazinon in aqueous solutions. Herein three aspects are focused on as follows:

(1) The synthesis of a new hybrid catalyst using three materials (nano-MgO, CNT and graphite), to enhance the stability, reusability and easy separation of the catalyst.

(2) The investigation of the removal and mineralization efficiency of diazinon *via* a COP.

(3) The exploration of the diazinon degradation mechanism *via* a COP.

RSM was used for the optimization study, and BBD was used to determine the optimum parameters for the catalytic ozonation of diazinon and also to explain the interactions between the studied parameters.

## 2. Materials and methods

### 2.1. Materials

All the chemicals used in this study were obtained from Merck (Germany). Only analytical grade chemicals were used. The MgO nanoparticles were prepared *via* a precipitation method.^39,40^ Diazinon was purchased from Sigma-Aldrich (Germany) and was of analytical grade. All solutions were made with ultra-pure water (with an electrical resistance of 18 μΩ cm at 25 °C). Diazinon stock solution (40 mg L^−1^) was prepared and stored in a refrigerator for further use. The CNTs (>95% carbon) were obtained from the Neutrino Company (Tehran, Iran). Graphite (≥97 wt%) was purchased from Sinochem (Tehran, Iran). Purified kaolinite (Al_2_Si_2_)_5_(OH)_4_ (≥90%) was purchased from Iran China Clay Industries Co. Real sewage samples were taken from the storage tanks of a pesticide manufacturing plant and used after 10-fold dilution and filtration using membranes with a pore diameter of 0.45 μm (PTFE, Membrane Solutions).

### 2.2. Catalyst preparation

For synthesizing the magnesium nanoparticles, 0.2 M magnesium nitrate (MgNO_3_·6H_2_O) (98 wt%) was mixed into 100 mL of deionized water in a 1 L beaker. Afterward, 0.4 M sodium hydroxide was mixed into 100 mL of de-ionized water and this was then added to the prepared magnesium nitrate solution in a dropwise fashion. The solution was stirred for 4 h at 250 rpm to separate the magnesium hydroxide. The obtained substance was centrifuged at 3500 rpm for 10 min, and Mg(OH)_2_ gel was obtained. The obtained gel was washed several times with methanol and de-ionized water, dried in an oven (FG-BF400E) at 90 °C for 48 h, and then calcified at 450 °C for 4 h.^41,42^

For preparing the proposed composite, the raw materials (MgO nanoparticles, CNTs, and graphite: 81.5%, 14% and 4.5% by weight, respectively), kaolinite (up to 10% by weight of the raw materials), and distilled water (as needed) were mixed in a stainless steel cup using a planetary mill (NARYA-MPM 4*250) to create a homogeneous mixture. The obtained slurry was dried at 120 °C for 48 h. The resulting composite was sintered in an electrical furnace with a corundum furnace tube (60 mm in diameter, 1000 mm in length; AZAR Furnaces, TF5/40-1720) under a hydrogen- and argon-controlled atmosphere for 2 h at 1350 °C to create a solid compound. The prepared composite was crushed into fine granules (40–30 mesh). The obtained granules were washed two times with de-ionized water, dried at 90 °C for 48 h, and stored in a desiccator for use in further experiments.

### 2.3. Ozonation procedure

Ozone was produced using an ozone generator (ARDA, MOG-5G-H) with a pure oxygen (≥99.999%) gas feed. The ozone was continually introduced into a Pyrex cylindrical impinger, acting as the reactor, with a volume of 500 mL, which was operated in semi-batch mode with a constant O_3_ gas flow rate of 3 L min^−1^ (1 g h^−1^ ≈ 16.7 mg min^−1^). The reactor exhaust gas was treated with 2% KI solution (see Fig. S1†). The diazinon removal experiments were conducted with 500 mL of reaction solution at the desired concentration; the preset amount of catalyst was added, and ozonation was then carried out for the specified time. The solution pH was adjusted using NaOH or HCl normal solutions at the desired level. All experiments were performed at 23 ± 3 °C (room temperature). The catalyst was separated from solution after 1 min of simple sedimentation. Residual dissolved ozone was quenched using Na_2_S_2_O_2_. The sample was filtered using a 0.2 μm PTFE syringe filter to separate probable catalyst particles, then the filtrate was analyzed for diazinon and total organic carbon (TOC). Also, control experiments were carried out utilizing single ozonation with no catalyst (SOP). The efficiencies of single ozonation and catalytic ozonation were compared under the desired conditions. The diazinon degradation efficiencies from the single ozonation and catalytic ozonation process experiments were calculated using the following equation:^43^1
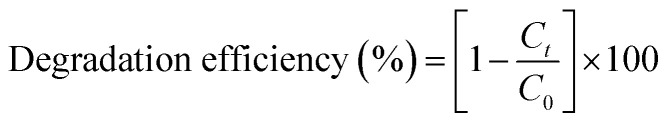
where *C*_0_ and *C*_*t*_ indicate the initial and ultimate diazinon concentrations, respectively, of the solution from a specific experiment.

The degree of mineralization of diazinon in the SOP and COP experiments was calculated according to eqn (2):^44^2
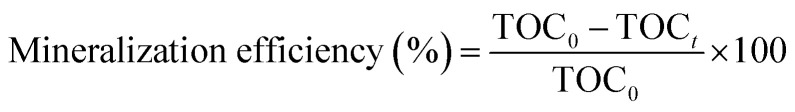
where TOC_0_ and TOC_*t*_ indicate the initial and ultimate TOC, respectively, of the solution from a target experiment.

The catalytic activity of the synthesized catalyst was obtained as follows:^9^

3 Catalytic activity (%) = [RE_COP_ − (RE_SOP_ + RE_ads_)]

where RE_COP_, RE_SOP_, and RE_ads_ are the diazinon removal efficiencies using nano-MgO@CNT@Gr/O_3_, the single ozonation process, and adsorption *via* nano-MgO@CNT@Gr without O_3_.

### 2.4. Response surface methodology

Design-Expert software (version 7.0.0) was used for statistical analysis. Box–Behnken experimental design with RSM was utilized to specify the independent parameters and to optimize the experimental parameters for diazinon removal *via* the catalytic ozonation process.^45^ Four independent variables, pH (*X*_1_; 3–10), catalyst loading (*X*_2_; 0.5–1.5 g L^−1^), ozonation time (*X*_3_; 5–15 min), and diazinon concentration (*X*_4_; 5–15 mg L^−1^), were fixed with low, medium, and high levels, which were coded as −1, 0 and +1 (Table 1). Each independent parameter level was determined according to eqn (4) as follows:^44,45^4
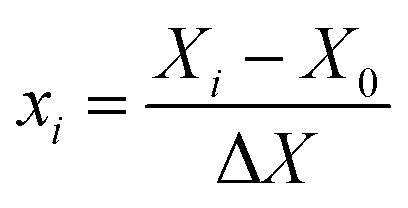
where *x*_*i*_ is the coded value, *X*_0_ is the real value of the independent parameter at the center point, *X*_*i*_ is the real value of the independent parameter, and Δ*X* is the step change value between low and high levels.

**Table 1:** The experimental values and levels for the design of the experiments

Independent process variables	Code	Real values of coded levels		
		−1	0	1
pH	*X*_1_	3	7	10
Catalyst loading	*X*_2_	0.5	1	1.5
Time	*X*_3_	5	10	15
Diazinon concentration	*X*_4_	5	10	15

According to BBD, the experimental design included five replicates of the central points and 29 factorial points (see Table 2).

**Table 2:** The experimental designs and RSM results

Std	Run	*X*_1_	*X*_2_	*X*_3_	*X*_4_	Actual value	Predicted value	*ε*
21	1	3	0.5	10	10	67.90	66.55	1.35
10	2	3	1	5	10	67.56	67.78	−0.23
1	3	7	1.5	15	10	74.70	76.60	−1.90
2	4	3	1.5	10	10	72.89	73.88	−0.99
29	5	7	1	10	10	72.18	70.19	1.98
17	6	10	1	10	5	74.92	75.46	−0.54
16	7	7	0.5	10	5	63.70	66.19	−2.49
8	8	3	1	15	10	75.12	73.51	1.62
11	9	10	1.5	10	10	76.35	77.24	−0.89
4	10	3	1	10	15	65.44	65.75	−0.31
6	11	7	1	10	10	70.80	70.19	0.60
28	12	7	1	15	5	75.56	73.72	1.84
5	13	7	1	5	5	66.43	64.80	1.63
25	14	7	1.5	10	5	72.48	71.47	1.01
22	15	7	1.5	5	10	69.54	69.09	0.46
26	16	7	1	10	10	70.26	70.19	0.07
9	17	7	0.5	5	10	65.16	63.93	1.24
12	18	10	1	15	10	80.10	79.75	0.35
14	19	7	0.5	10	15	59.68	60.87	−1.19
19	20	10	1	5	10	69.37	71.47	−2.11
15	21	7	1	5	15	60.43	61.43	−0.99
18	22	7	1.5	10	15	68.88	66.58	2.31
7	23	3	1	10	5	66.45	67.89	−1.44
13	24	7	0.5	15	10	69.66	70.78	−1.12
20	25	7	1	10	10	69.67	70.19	−0.52
24	26	10	0.5	10	10	75.35	73.13	2.22
3	27	10	1	10	15	69.10	68.13	0.97
23	28	7	1	10	10	68.06	70.19	−2.14
27	29	7	1	15	15	66.09	66.88	−0.79

Experiments were performed based on the design points and data were assessed to determine the contributions of the four parameters, which were then correlated using a second-order polynomial equation as follows:^44,45^5
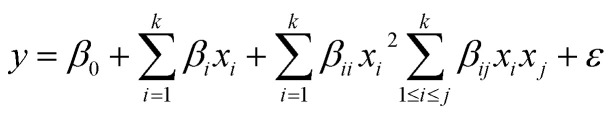
where *y* signifies the predicted response; *x*_*i*_ and *x*_*j*_ represent the coded independent parameters, *β*_0_ is a constant coefficient, *β*_*i*_, *β*_*ii*_ and *β*_*ij*_ are regression coefficients for linear, quadratic and interaction effects, respectively, *ε* is the random error, and *k* is the number of desired independent variables.

For analysis of the results, Design-Expert software was used and ANOVA was used for quadratic and interaction term determination.^46^

### 2.5. Characterization of the optimum catalyst

The chemical composition of nano-MgO/CNT/graphite was analyzed *via* X-ray diffraction (XRD) (PW1730 – Philips), scanning over 2*θ* values between 10° and 80°. The functional groups on the surface of the catalyst were analyzed *via* FTIR spectroscopy (Avatar, Thermo) over wavenumbers ranging from 4000 to 400 cm^−1^. The results of EDX microanalysis were used to show the existence of primary components in the synthesized catalyst. Microstructural evaluation was performed using FESEM (FE-SEM, Mira III, Czech Republic) equipped with energy dispersive X-ray (EDX) microanalysis to characterize the surface structure of the catalyst. Specific surface area (SSA) and pore size measurements of the catalyst particles were carried out *via* the Brunauer–Emmett–Teller (BET) method using N_2_ adsorption/desorption data obtained using BELSORP mini II apparatus (Japan) at 77 K according to the BET and Barrett–Joyner–Halenda (BJH) isotherm models. The pH of the point of zero charge (pH_PZC_) was determined *via* the conventional method.^39^

### 2.6. Analytical methods

The potassium iodide (KI) method was used for determining the utilized ozone dosage.^47^ The diazinon concentration was measured *via* HPLC (Agilent 1260, Santa Clara, CA) using a C18 column (10 cm length and an inside diameter of 4.6 mm, Eclipse Plus, Agilent) with a UV detector at 254 nm. A 65 : 35 (v/v) mixture of acetonitrile and water was used as the mobile phase at a flow rate of 1.0 mL min^−1^. TOC measurements were carried out using a TOC analyzer (Shimadzu, TOC analyzer, VCSH model). The COD concentration measurements were performed using the closed reflex method.^47^ Intermediate analysis was performed using GC/MS apparatus (Agilent 6890-5973N) equipped with a HP-5 MS capillary column (30 m length, 0.25 mm I.D., 0.25 μm thickness). Helium at a constant flow rate of 1 mL min^−1^ was used as the carrier gas. The MS interface temperature was 280 °C and the ion source temperature was 230 °C. The oven temperature program was as follows: 1.2 min at 40 °C; 4.2 °C min^−1^ to 50 °C; 16.7 °C min^−1^ to 80 °C (1.2 min).

## 3. Results and discussion

### 3.1. Catalyst characterization

**Fig. 1:**
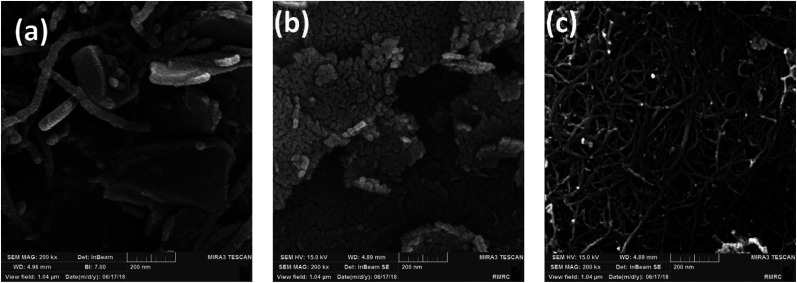
FESEM images of (a) the nano-MgO@CNT@Gr composite, (b) nano-MgO powder and (c) CNT powder.

An FESEM image of the nano-MgO@CNT@Gr composite is shown in Fig. 1a. In the FESEM image of the composite, it is evident that the shapes and sizes of the nano-MgO particles and CNTs, as seen in Fig. 1b and c, were preserved. Furthermore, it is quite clear that the composite has a heterogeneous and irregular shape and a porous-like surface. Using the method we describe, all components were successfully combined uniformly with each other.

**Fig. 2:**
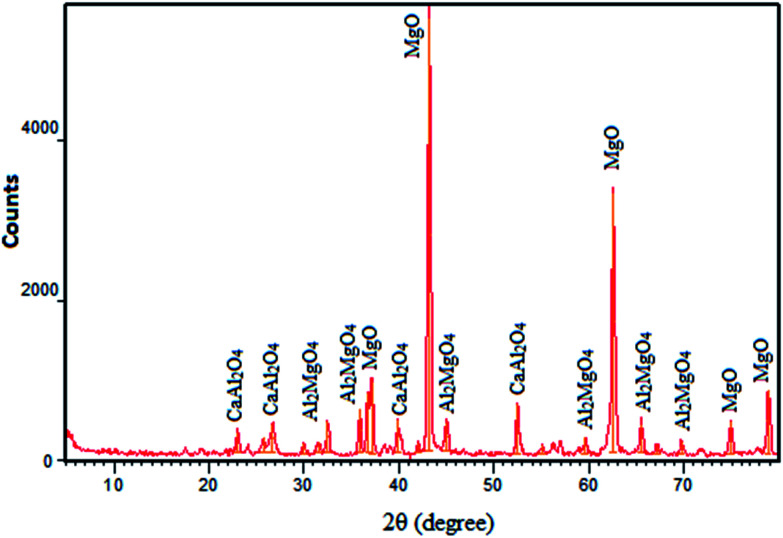
The nano-MgO@CNT@Gr composite XRD pattern.

The synthesized nano-MgO@CNT@Gr composite was characterized *via* X-ray diffraction (XRD). The XRD pattern is shown in Fig. 2. Sharp peaks at 2*θ* values of 37.17°, 43.17°, 62.59°, 74.93° and 78.86° are associated with MgO, and weak peaks observed at other 2*θ* values are associated with Al_2_MgO_4_, Al_0.58_Mg_0.42_, Al_2_O_3_, and CaAl_2_O_4_. These results match the major peaks of periclase MgO given by the Joint Committee on Powder Diffraction Standards card no. 45-0946.^15^

The mean crystallite size (*D*) of nano-MgO@CNT@Gr particles was calculated using the Debye–Scherrer formula^26,39,41^ given by eqn (6):6
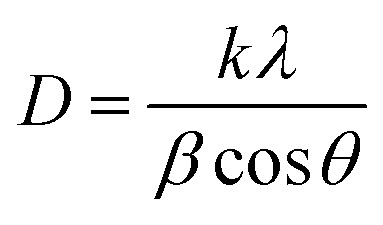
where *D* is the particle size, *λ* is the X-ray wavelength of Cu Kα radiation (1.5418 Å), *k* is the shape factor (0.9), *β* is the full width at half maximum (FWHM), and *θ* is the angle of reflection. The average size of the nano-MgO@CNT@Gr crystals was calculated to be 22.65 nm. The crystalline size of nano-MgO@CNT@Gr prepared in this study is close to the crystalline size of MgO reported in the literature.^15,39,48^ Based on the results of XRD analysis, MgO nanoparticles were shown to be successfully coated on the CNTs and graphite.

**Fig. 3:**
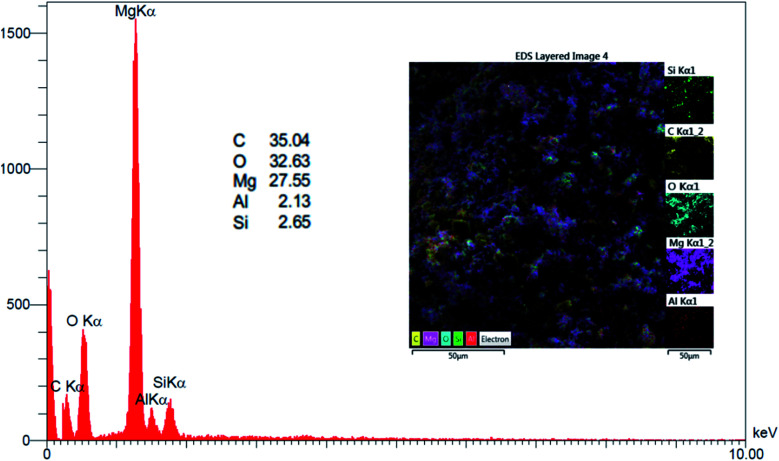
EDX analysis of nano-MgO@CNT@Gr.

EDX analysis (Fig. 3) confirmed that nano-MgO@CNT@Gr is made up of magnesium (Mg; 27.55%), carbon (C; 35.04%), oxygen (O; 32.63%), and small amounts of aluminum (Al; 2.13%) and silica (Si; 2.65%). Therefore, all the components of nano-MgO@CNT@Gr have been preserved during the synthesis process.

**Fig. 4:**
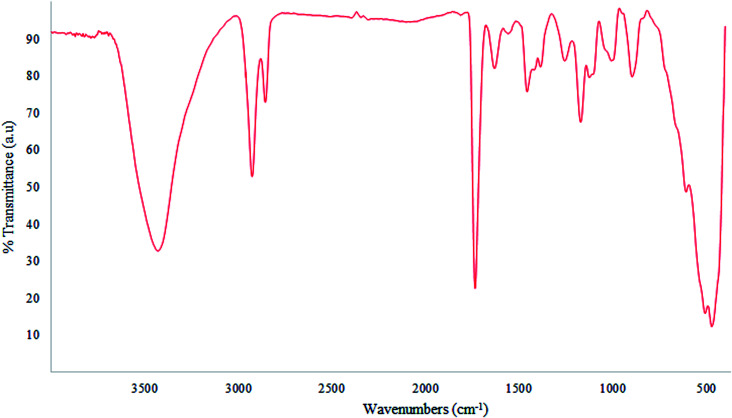
The FTIR spectrum of nano-MgO@CNT@Gr.

From the FTIR spectrum of the nano-MgO@CNT@Gr composite (Fig. 4), the strong broad band at 3430 cm^−1^ can be attributed to the stretching mode of surface hydroxyl groups (O–H) present on the surface due to the physical adsorption of molecular water. The peaks at 1630 and 1735 cm^−1^ are related to the bending mode of these hydroxyl groups.^48^ The wide band at 1457 cm^−1^ and band at 600 cm^−1^ can be assigned to the Mg–O stretching mode. The peak at 1171 cm^−1^ is associated with H^−^ ions.^39^ Therefore, it was deduced that the surface of nano-MgO@CNT@Gr is covered mainly in hydroxyl groups.

Based on the BET surface area and total pore volume results, the surface area of nano-MgO@CNT@Gr was calculated to be 221.631 m^2^ g^−1^. The total pore volume of nano-MgO@CNT@Gr (at *P*/*P*_0_ = 0.99) was found to be 0.155 cm^3^ g^−1^, with an average pore size of 3.65 nm. This shows, therefore, that nano-MgO@CNT@Gr prepared *via* the selected procedure is a mesoporous material (as per IUPAC classifications) with a high SSA. The SSA is one of the main parameters affecting the catalytic activity of a substance, due to the provision of available sites for the adsorption of pollutants and/or the production of reactive species. Hence, preparing catalysts with a high SSA is one of the main points being focused on by researchers in this field.^39^ Thus, it was expected that the synthesized nano-MgO@CNT@Gr would have high catalytic activity.

### 3.2. Model fitting

Based on four-factor three-level BBD, 29 experiments were carried out to obtain the efficient removal of diazinon and determine the interactions between experimental factors, including five replications at the center point. Modeling of the quadratic polynomial equation between the response and independent variables was applied, which includes pH (*X*_1_), catalyst loading (*X*_2_), ozonation time (*X*_3_), and diazinon concentration (*X*_4_). For optimized RSM, a sufficient model fit should be gained to obtain rich and clear results; this is necessary to ensure the adequacy of the model. Table 1 presents the RSM factors and levels. The RSM experimental design and results are presented in Table 2. Variance analysis (ANOVA) of the model regression parameters for the removal of diazinon *via* a COP using the results from the carried-out experiments are shown in Table 3. The ANOVA results for the second-order quadratic regression indicate the higher significance of the model. The model *F*-value is 10.21, with a *p*-value of less than 0.0001. This *p*-value of less than 0.05 indicates that the model terms for diazinon removal are significant; it shows that only 0.01% of the total variation could not be explained by the model due to noise. The lack of fit is the variation of the data around the fitted model and tests the adequacy of a model fit. The “lack of fit *F*-value” of 2.07 shows that the lack of fit was not meaningful relative to the pure error, and this model successfully fitted the data.

**Table 3:** ANOVA results from the response surface quadratic model for diazinon removal

Regression	d.f.	Sum of squares	Mean square	*F*-value	Pr > *F*
Linear	20	245.84	12.29	5.38	0.0570
Quadratic	10	47.32	4.73	2.07	0.2516
Cubic	2	2.37	1.19	0.52	0.6302
Pure error	4	9.14	2.28		

The *R*^2^ value of 0.9108 suggests that the empirical model could not describe only 8.92% of the total variation. This demonstrated the sufficiency of the quadratic fit to navigate the design space. Therefore, the RSM developed in the present study for diazinon removal efficiency prediction was considered to be appropriate.

A small gap between *R*_adj_^2^ and *R*^2^ is acceptable for the judgment of the model correctness. The calculated *R*_adj_^2^ of 0.8215 means that the independent variables did not describe 17.85% of the total variation. The variation coefficient (CV) indicates the model reproducibility and is represented as the percentage ratio between the standard error of the estimate and the mean observed response. A model CV of less than 10% can be considered as reasonably reproducible.^49^ The model “Adequate Precision” measures the range of predicted values and their related error. The model CV was 2.88 (less than 10%), and the adequate precision value was 13.074 (higher than 4), implying that the model fitness was desirable.

The ANOVA of the quadratic model coefficients for diazinon removal are shown in Table 4. The table shows that six terms (*X*_1_, *X*_2_, *X*_3_, *X*_4_, *X*_1_^2^, and *X*_4_^2^) were judged to be statistically meaningful for diazinon removal. The model independent variables pH (*X*_1_), catalyst loading (*X*_2_), reaction time (*X*_3_), and diazinon concentration (*X*_4_), and the quadratic terms *X*_1_^2^ and *X*_4_^2^ were highly considerable based on a 95% confidence level (*p* < 0.05). The order of the *F*-values of the four factors is as follows: time (*X*_3_) > catalyst loading (*X*_2_) > pH (*X*_1_) > diazinon concentration (*X*_4_), and it appears that time (*X*_3_) has most considerable impact on diazinon decomposition. The actual values and predicted values of diazinon removal efficiency were close to the same straight line (see Fig. S2†). This denotes that the predicted values attained from the model were in acceptable accordance with the obtained experimental data. As anticipated, pH, catalyst loading, and reaction time had significant positive effects, while the concentration of diazinon had a considerable negative impact.

**Table 4:** The response surface second-order model ANOVA results for the removal of diazinon^a^

Source	Sum of squares	d.f.^b^	Mean square	*F*-value	*P*-value	
Model	576.15	14	41.15	10.21	<0.0001	Significant
*X*_1_	74.06	1	74.06	18.37	0.0008	
*X*_2_	95.62	1	95.62	23.71	0.0002	
*X*_3_	143.25	1	143.25	35.52	<0.0001	
*X*_4_	65.52	1	65.52	16.25	0.0012	
*X*_1_ *X*_2_	2.61	1	2.61	0.65	0.4346	
*X*_1_ *X*_3_	1.66	1	1.66	0.41	0.5318	
*X*_1_ *X*_4_	6.82	1	6.82	1.69	0.2143	
*X*_2_ *X*_3_	0.11	1	0.11	0.027	0.8710	
*X*_2_ *X*_4_	0.046	1	0.046	0.011	0.9169	
*X*_3_ *X*_4_	3.01	1	3.01	0.75	0.4020	
*X*_1_^2^	62.64	1	62.64	15.53	0.0015	
*X*_2_^2^	0.44	1	0.44	0.11	0.7454	
*X*_3_^2^	0.18	1	0.18	0.044	0.8368	
*X*_4_^2^	86.65	1	86.65	21.49	0.0004	
Residual	56.46	14	4.03			
Lack of fit	47.32	10	4.73	2.07	0.2516	Not significant
Pure error	9.14	4	2.28			
Corrected total	632.61	28				

a *R* ^2^ = 0.9108, *R*_adj_^2^ = 0.8215, *R*_pred_^2^ = 0.5475.

b Degrees of freedom.

The final quadratic polynomial regression equation in terms of the coded variables is presented in eqn (7).

7 *Y* (%) = +69.77 + 2.48*X*_1_ + 2.86*X*_2_ + 3.5*X*_3_ − 2.37*X*_4_ − 0.80*X*_1_*X*_2_ + 0.64*X*_1_*X*_3_ − 1.30*X*_1_*X*_4_ − 0.17*X*_2_*X*_3_ + 0.11*X*_2_*X*_4_ − 0.87*X*_3_*X*_4_ + 3.19*X*_1_^2^ − 0.26*X*_2_^2^ + 0.17*X*_3_^2^ − 3.65*X*_4_^2^ (subject to −1 ≤ *X*_*i*_ ≤ +1)

where negative and positive values represent decreases and increases in the response, respectively. Following eqn (7) the variables *X*_1_, *X*_2_, and *X*_3_ had positive impacts and the variable *X*_4_ harmed diazinon removal *via* the COP.

An empirical relationship between the diazinon removal efficiency and the variables is presented *via* the following quadratic polynomial equation:

8 *Y* (%) = +45.36669 − 1.84074*X*_1_ + 9.69894*X*_2_ + 0.61102*X*_3_ + 3.23677*X*_4_ − 0.45843*X*_1_*X*_2_ + 0.036540 *X*_1_*X*_3_ − 0.074137*X*_1_*X*_4_ + 0.066438*X*_2_*X*_3_ + 0.042664*X*_2_*X*_4_ − 0.034710*X*_3_*X*_4_ + 0.26038*X*_1_^2^ − 1.04458*X*_2_^2^ + 6.61826*E* − 003*X*_3_^2^ − 0.14620*X*_4_^2^ (subject to: 3 ≤ pH ≤ 10; 0.5 g L^−1^ ≤ catalyst loading ≤ 1.5 g L^−1^; 5 min ≤ time ≤ 15 min; and 5 mg L^−1^ ≤ diazinon concentration ≤ 1.5 mg L^−1^)

### 3.3. Analysis of the response surface

Three-dimensional curves of the response surfaces and two-dimensional contour plots were obtained to determine the optimal values of the variables. The model response surfaces and contour plots were obtained with two variables held at the central level and the other two variables varied in the experimental ranges. The results are shown in Fig. S3–S8.† In Fig. S3,† the response surface and contour plot were obtained as a function of pH and catalyst loading. At the same time, the time and diazinon concentration were kept constant at 10 min and 10 mg L^−1^, respectively. As shown in Fig. S3,† a higher diazinon removal efficiency (76%) was realized at 10 min and 10 mg L^−1^ diazinon concentration when the pH and catalyst loading variables were at higher values (10 and 1.5 g L^−1^, respectively).

The impact of the pH and time variables on the diazinon removal efficiency is demonstrated in Fig. S4.† When the diazinon concentration and catalyst loading were kept fixed at 10 mg L^−1^ and 1 g L^−1^, respectively, the diazinon removal efficiency increased with increasing time and pH. The relationship between the factors, hence, was significant in terms of optimization; however, the time and pH had a more significant effect on the efficiency of diazinon removal.

To investigate the impact of pH and diazinon concentration on diazinon removal, tests were carried out at diazinon concentrations varying from 5 to 15 mg L^−1^ at various pH levels at a fixed catalyst loading of 1 g L^−1^ and time of 10 min (see Fig. S5†). The figure displays that the removal efficiency of diazinon increased with rising pH at the central level of diazinon concentration.

The impact of time and catalyst loading on the removal efficiency of diazinon is presented in Fig. S6.† When pH and diazinon concentration was kept fixed at 7 and 10 mg L^−1^, respectively, the diazinon removal efficiency increased with increasing time and catalyst loading. Therefore, the time and catalyst loading (due to an increased surface area) have a more considerable effect on the diazinon removal efficiency.

As shown in Fig. S7,† the catalyst loading and diazinon concentration were also varied. At the same time, the time and pH were kept constant at central levels. The diazinon removal efficiency increased with increased catalyst loading at the central level of diazinon concentration.

As shown in Fig. S8,† 73% diazinon removal efficiency was realized at the highest application time and with the diazinon concentration at the central level, while the pH and catalyst loading levels were kept fixed at 7 and 1 g L^−1^, respectively.

### 3.4. Validation of the model

According to the results obtained from the response surface model, the maximum theoretical removal efficiency of diazinon was attained under the optimal conditions as follows: pH, 10; catalyst loading, 1.5 g L^−1^; time, 15 min; and diazinon concentration: 10 mg L^−1^. To investigate the reliability of the RSM, a series of additional experiments were performed in triplicate under the optimum conditions. An average diazinon removal efficiency of 82.43% was obtained from the experiments. The experimental value was in good agreement with the predicted maximum diazinon removal efficiency from the quadratic model, which indicates that the model can better express the experimental conditions. Through using a response surface model to optimize the experimental factors for diazinon removal, the accuracy and validity of the model approach and the reliability of RSM could be confirmed.

### 3.5. Catalytic mechanism

The pH of the solution plays an essential role in catalytic ozonation, as it can affect the catalyst surface properties, the dissociation constant (p*K*_a_), and the production of active radicals.^25^ The pH_PZC_ value of synthesized nano-MgO@CNT@Gr in these experiments was 9.3 (data not shown). Thus nano-MgO@CNT@Gr is considered a strongly basic oxide substance with a high isoelectric point.^39^ Therefore, it was expected that the surface of nano-MgO@CNT@Gr would be covered broadly by hydroxyl groups.^39^ This is in close agreement with the strong broad band from OH observed in the FTIR spectrum. Surface hydroxyl groups are active sites for hydroxyl radical production.^50^ Therefore, the larger the number of active sites, the higher the production of hydroxyl radicals; thereby, the better removal of organic matter is achieved. The predominance of hydroxyl groups on the surface of the catalyst can be related to surface hydroxylation due to the dissociative chemisorption of water molecules.^51^ In summary, the synthesized nano-MgO@CNT@Gr compound consists of mesoporous nanoparticles with a very high specific surface area and a high density of basic surface functional groups. These features are firmly favorable for an ozonation catalyst.

**Fig. 5:**
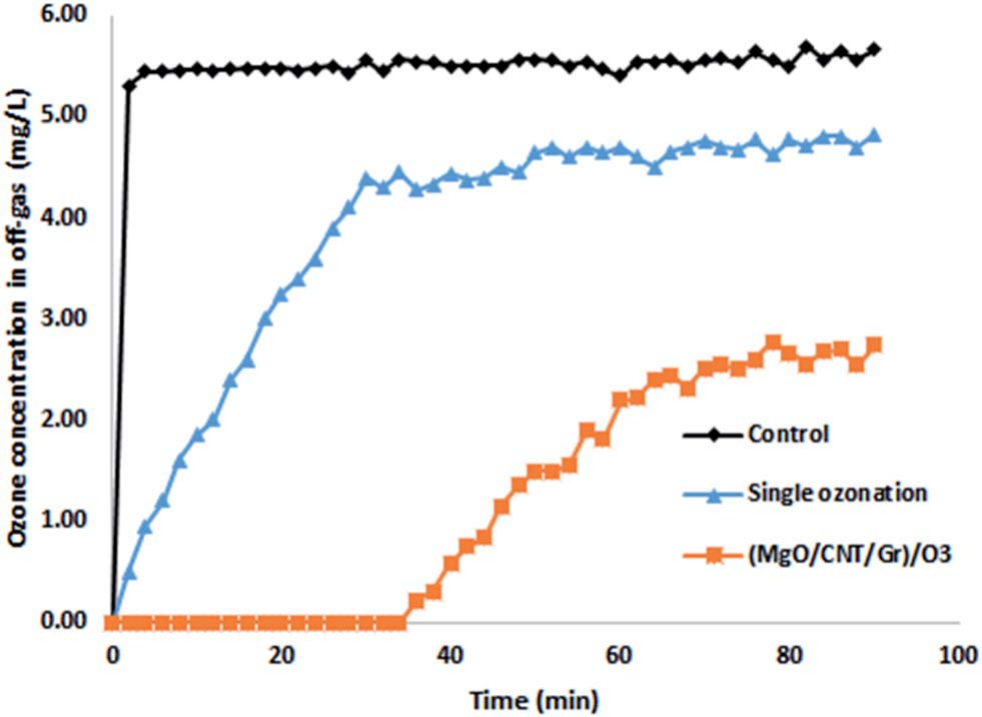
The off-gas ozone concentration (O_3_ flow rate: 16.7 mg min^−1^; nano-MgO@CNT@Gr loading: 1.5 g L^−1^; diazinon concentration: 10 mg L^−1^; pH: 10).

Also, the O_3_ concentrations in the inlet gas and off-gas were measured during the reaction (see Fig. 5). The former was controlled at 5.5 ± 0.2 mg L^−1^. The off-gas O_3_ concentration in the control group experiment (deionized water without diazinon or catalyst) immediately increased to 5.52 mg L^−1^ after 4 min, which indicated that O_3_ quickly saturated the solution.

In the single ozonation process, the O_3_ concentration in the off-gas gradually increased and then it reached a steady-state within 30 min of about 4.6 mg L^−1^. This result shows that the aqueous O_3_ was consumed constantly. Following Henry's law, due to the consumption of O_3_, the solution remained unsaturated, so O_3_ was continuously transferred from the gas phase to the liquid phase. In the COP, at first, the off-gas O_3_ concentration remained at zero, but after 35 min it started to rise. The absence of O_3_ in the off-gas indicated that O_3_ was consumed immediately after being transferred to the liquid phase. Similar to the single ozonation process, the ozone concentration in the effluent gradually increased in the presence of nano-MgO@CNT@Gr. These results demonstrate that nano-MgO@CNT@Gr can enhance O_3_ degradation.

**Fig. 6:**
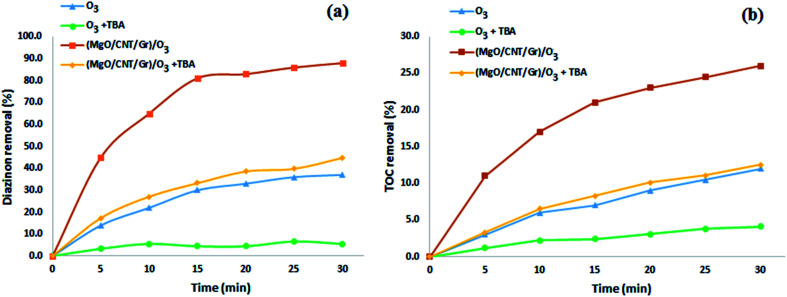
(a) Diazinon and (b) TOC removal rates during the SOP and COP (O_3_ flow rate: 16.7 mg min^−1^; catalyst loading: 1.5 g L^−1^; diazinon concentration: 10 mg L^−1^; pH: 10).

Organic substance reactions directly with O_3_ and indirectly with ˙OH are typically observed during heterogeneous catalytic ozonation.^19^*tert*-Butanol (TBA) as a conventional scavenger can react with hydroxyl radicals more selectively with a higher reaction rate constant of 6 × 10^8^ M^−1^ s^−1^ while reacting with O_3_ at a rate constant of 3 × 10^−3^ M^−1^ s^−1^.^19^ Therefore, to identify the presence of ˙OH in the reactions, TBA was broadly applied as a probe molecule. To study the effects of nano-MgO@CNT@Gr on the generation of ˙OH, 2 g (4 g L^−1^) of TBA was added to the reactor. As seen in Fig. 6, TBA addition to the reaction decreased diazinon removal in both the single ozonation and nano-MgO@CNT@Gr/O_3_ processes. This shows the presence of ˙OH radicals in the nano-MgO@CNT@Gr/O_3_ process. Also, the effects of nano-MgO@CNT@Gr on O_3_ decomposition and ˙OH generation were investigated.

As stated above, the addition of nano-MgO@CNT@Gr to the ozonation process catalyzes the oxidation of diazinon *via* the enhancement of ozone decomposition, so leading to the production of oxidative radical species in the form of ˙OH. Catalytic ozonation involves two mechanisms in reactions with organic substances: direct reactions with ozone and indirect reactions with produced radicals. Reactions can occur both at the surface of the catalyst and in solution. The proposed mechanisms can be described as follows:

• O_3_ direct oxidation in solution:

9 O_3_ + diazinon → CO_2_ + H_2_O + intermediate

• Radical oxidation in solution:

10 ˙OH + diazinon → CO_2_ + H_2_O + intermediate

• O_3_ direct oxidation on the catalyst surface:

11 (nano-MgO@CNT@Gr)^−O_3_^ + diazinon → CO_2_ + H_2_O + intermediate

12 (nano-MgO@CNT@Gr)^−diazinon^ + O_3_ → CO_2_ + H_2_O + intermediate

• Radical oxidation on the catalyst surface:

13 (nano-MgO@CNT@Gr)–S + O_3_ → (nano-MgO@CNT@Gr)–S^O=^O–O^^ → MgO^−S^O˙^^ + O_2_

14 (nano-MgO@CNT@Gr)–S^O˙^ + 2H_2_O + O_3_ → (nano-MgO@CNT@Gr)–S^(˙OH)_2_^ + 3˙OH + 2O_2_

15 (nano-MgO@CNT@Gr)–S^(˙OH)_2_^ + diazinon → CO_2_ + H_2_O + intermediate

16 (nano-MgO@CNT@Gr)^−diazinon^ + ˙OH → CO_2_ + H_2_O + intermediate

where S in (nano-MgO@CNT@Gr)–S represents Lewis acid sites on the nano-MgO@CNT@Gr particle surface.

According to the results from TBA experiments, it can be concluded that ˙OH indirect reactions are the major mechanism involved in diazinon degradation in the COP, as shown in the reaction presented in eqn (16). pH analysis demonstrated that the surface hydroxyl groups played a significant role in the diazinon catalytic ozonation process. The results showing the dissolved ozone variations demonstrated that the catalyst could enhance the production of hydroxyl radicals. Due to the low diazinon adsorption (around 2.77%) on the catalyst, another probable mechanism based on eqn (16) could be somewhat involved.

The removal of diazinon during single nano-MgO@CNT@Gr adsorption was about 2.77%, which is negligible and indicates that using nano-MgO@CNT@Gr as an adsorbent for the adsorption removal of diazinon is not viable. As shown in Fig. 6, about 88% of diazinon and 26% of the TOC was removed using nano-MgO@CNT@Gr/O_3_ after 30 min, whereas the diazinon and TOC removal values *via* single ozonation were about 37% and 12% after the same time period, respectively. These results indicate that nano-MgO@CNT@Gr is highly effective for the COP. To better explain the catalytic activity of nano-MgO@CNT@Gr in the catalytic ozonation process, the kinetics of diazinon removal were compared according to the following reaction model (eqn (17) and (18)):^52^17
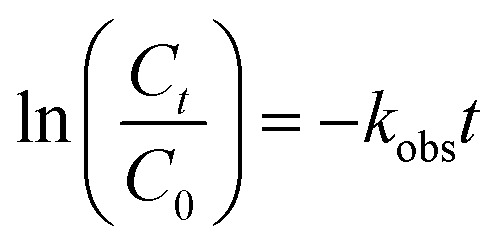
18
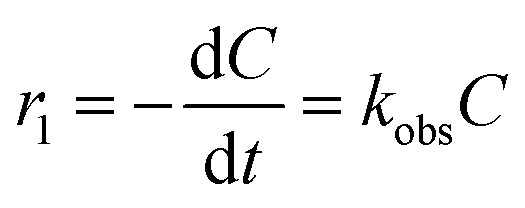
in which C_0_ and *C*_*t*_ indicate the initial and the residual amounts of diazinon, respectively, and *k*_obs_ is the PFO reaction rate constant. According to the results, the PFO model shows a desirable fit (*R*^2^ > 0.93) with the diazinon removal efficiency. The pseudo-first-order rate constant of nano-MgO@CNT@Gr/O_3_ (0.0079 min^−1^) was almost 4.6 times higher than that obtained *via* single ozonation (0.0017 min^−1^) (see Table 5).

**Table 5:** Kinetic information relating to diazinon removal *via* O_3_ and nano-MgO@CNT@Gr/O_3_

	*R* ^2^	*k*_obs_ (min^−1^)	*r* (mg L^−1^ min^−1^)	*r*_COP_/*r*_SOP_
O_3_	0.92	0.015	0.0017	4.59
Nano-MgO@CNT@Gr/O_3_	0.93	0.071	0.0079	

### 3.6. The degradation pathway

**Fig. 7:**
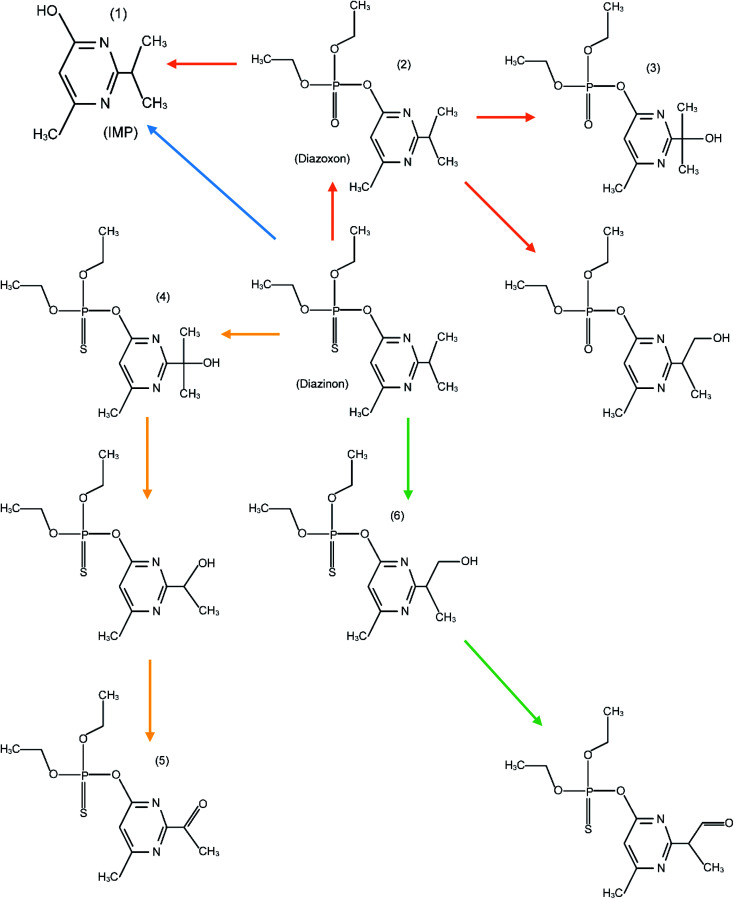
A schematic diagram showing the proposed diazinon degradation pathways during the nano-MgO@CNT@Gr/O_3_ process. The numbers match those given in Table 6.

The high degradation of diazinon has been observed during ozonation processes. Ohashi *et al.* (1994) identified several diazinon intermediates, including 2-isopropyl-6-hydroxylmethylpyrimidine (IMP), diazoxon, and triethyl phosphate, during the ozonation process.^53^ As has been illustrated by other authors, the degradation of organic compounds in the COP does not happen right away to form CO_2_, but rather it occurs through the formation of long-lived by-products.^54^ In this study, for the determination of organic intermediates, a relatively slow catalytic ozonation process (16.7 mg O_3_ per L, diazinon concentration: 10 mg L^−1^, catalyst loading: 1.5 g L^−1^) was used, which allowed us to obtain slower kinetics and establish desirable conditions for the identification of by-products. GC/MS was used for the identification of the diazinon degradation by-products from the COP. Analysis of samples obtained from the nano-MgO@CNT@Gr/O_3_ process permitted the identification of six intermediate products that were considered transformation compounds. Data relating to the six by-products are presented in Table 6. As shown in Fig. 7, two probable degradation routes were considered. The first route includes the cleavage of the P=O (pyrimidine group) bond, yielding IMP (compound 1).^53,54^ Studies have shown that IMP is much less toxic than its parent diazinon.^7,55^ The oxidation of the P=S bond of diazinon *via* the substitution of sulfur by oxygen leads to the formation of diazoxon (compound 2). The double oxidation of diazinon results in the production of hydroxydiazoxon (compound 3). Besides, ˙OH attack can lead to the production of a hydroxy derivative of diazoxon. In the nano-MgO@CNT@Gr/O_3_ system, the loss of the pyrimidine group was probably due to oxidative desulfuration as a result of hydroxyl radical attack at the thiono group (–P=S–O). Diazoxon was produced following hydrolysis or *via* an oxidative mechanism affecting diazinon directly.^56,57^ These oxidative mechanisms have previously been observed in metabolic studies of most thiophosphates.^57^ In the second route, the thiophosphoric portion of diazinon was preserved. Hydroxydiazinon (compound 4) or 2-hydroxydiazinon (compound 6) were produced *via* the hydroxylation of the primary or secondary carbon atoms of the isopropyl group. Upon further oxidation, diazinon methyl ketone (compound 5) and diazinon aldehyde were formed. After the oxidation of the P=S bond of diazinon to a P=O bond, the oxygen analogue diazoxon was formed, leading to the production of the hydroxylated derivatives. In a previous study it was reported that hydroxydiazinon (4) generated, *via* a dehydration reaction involving the 1-hydroxy-isopropyl group, an isopropenyl-substituted compound, which was subsequently oxidized and decarboxylated to the hydroxyethyl derivative.^57^ Other degradation by-products were probably present in the catalytic ozonation system but were not identified *via* GC/MS because of its limited sensitivity and their low concentrations.

**Table 6:** The products identified *via* GC/MS during the catalytic ozonation degradation of diazinon

Product no.	Compound name	*R*_t_ (min)	M.W.	Characteristic ions (*m*/*z*)	Elemental composition
1	2-Isopropyl-6-hydroxylmethylpyrimidine (IMP)	25.9	152	152, 137, 109	C_8_H_12_N_2_O
2	Diazoxon	29.7	288	288, 152, 151, 137	C_12_H_22_N_2_O_4_P
—	Diazinon	30.3	304	304, 137, 152, 179	C_12_H_22_N_2_O_3_PS
3	Hydroxydiazoxon	31.8	304	304, 111, 153, 289	C_12_H_22_N_2_O_5_P
4	Hydroxydiazinon	32.2	320	320, 292, 178, 153	C_12_H_22_N_2_O_3_PS
5	Diazinon methyl ketone	33.5	304	304, 199, 180, 153	C_11_H_18_N_2_O_4_PS
6	2-Hydroxydiazinon	34.9	320	320, 195, 151, 122	C_12_H_22_N_2_O_4_PS

### 3.7. Reusability of the catalyst

**Fig. 8:**
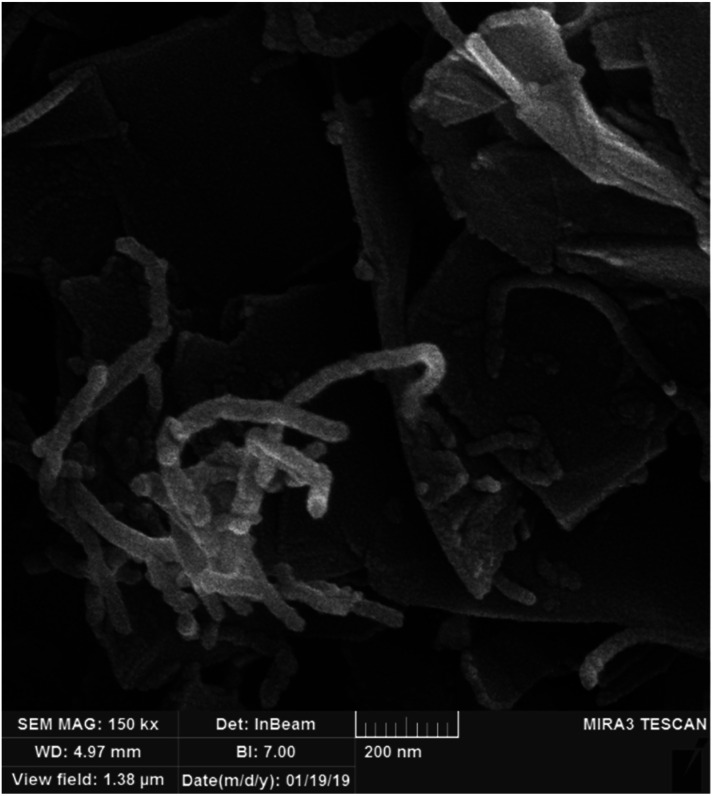
An FESEM image of nano-MgO@CNT@Gr after five sequential COP experiments.

Reusability and stability are major properties of a catalyst for its practical application in the degradation of pollutants. To evaluate the reusability of nano-MgO@CNT@Gr and the permanence of its catalytic activity, five sequential COP experiments were carried out under optimal experimental conditions: pH = 10; catalyst loading = 1.5 g L^−1^; diazinon concentration = 10 mg L^−1^; and time of reaction = 15 min. The catalyst was separated *via* simple sedimentation after each run. Then, the separated catalyst was washed with distilled water and dried for 24 h at 110 °C in an oven and reused for further COP experiments. The diazinon removal efficiency was calculated after each run, and the results are shown in Fig. S9.† The results showed that the diazinon removal efficiency for fresh nano-MgO@CNT@Gr was about 82.43% under the defined conditions. The diazinon removal efficiency was not significantly affected after five successive cycles of nano-MgO@CNT@Gr reuse, remaining at more than 78%. These results indicate that nano-MgO@CNT@Gr could maintain its catalytic capacity as well as its stability after five successive applications. Furthermore, the excellent chemical stability of nano-MgO@CNT@Gr was further confirmed *via* FESEM analysis of used and fresh catalyst samples (see Fig. 8). No considerable changes in the FESEM image were observed after five sequential COP experiments using the catalyst. This finding indicates that nano-MgO@CNT@Gr not only shows excellent catalytic activity but also acceptable stability.

## 4. Conclusions

This study investigated the effectiveness of a new hybrid catalyst that combines three materials, nano-MgO, CNTs, and graphite, for diazinon degradation during catalytic ozonation.

Box–Behnken experimental design and RSM were performed to specify the independent variables and optimize the experimental parameters of pH, catalyst loading, reaction time, and initial diazinon concentration. Regression analysis indicated the desirable fit of the experimental data with a second-order model with an *R*^2^ coefficient of >0.91. ANOVA of the coefficients for each independent variable showed that pH, catalyst loading, and reaction time had significant positive effects; in contrast, the diazinon concentration had a meaningful adverse impact on diazinon removal during catalytic ozonation. The optimum independent variable levels were found for maximum diazinon degradation (*i.e.*, 82.43% diazinon degradation): reaction time, 15 min; pH, 10; catalyst dose, 1.5 g L^−1^; and diazinon concentration, 10 mg L^−1^. The results showed that the nano-MgO@CNT@Gr/O_3_ process is a suitable technique for diazinon degradation. Six kinds of diazinon by-products were identified during the nano-MgO@CNT@Gr/O_3_ process. The diazinon degradation route was considered based on the distinguished intermediate products under the given set of reaction conditions. The advantages of the nano-MgO@CNT@Gr/O_3_ process as an oxidative treatment are its simple handling and rapid degradation. Thus, this catalytic ozonation process could be used for wastewater treatment as a newly developed method to reduce the levels of other pesticides and endocrine disrupting chemicals.

## Conflicts of interest

The authors declare no conflicts of interest.

## Supplementary Material

RA-010-C9RA10095D-s001
