# Supplementary material for: The catalytic ozonation of diazinon using nano-MgO@CNT@Gr as a new heterogenous catalyst: the optimization of effective factors by response surface methodology
Source: RSC Adv. 2020 Feb 21;10(13):7718–31. doi: 10.1039/c9ra10095d (PMC9049956; doi:10.1039/c9ra10095d)
Supplement: RA-010-C9RA10095D-s001 [file RA-010-C9RA10095D-s001.pdf]

Electronic Supplementary Material (ESI) for RSC Advances.
This journal is © The Royal Society of Chemistry 2020

Supplementary:

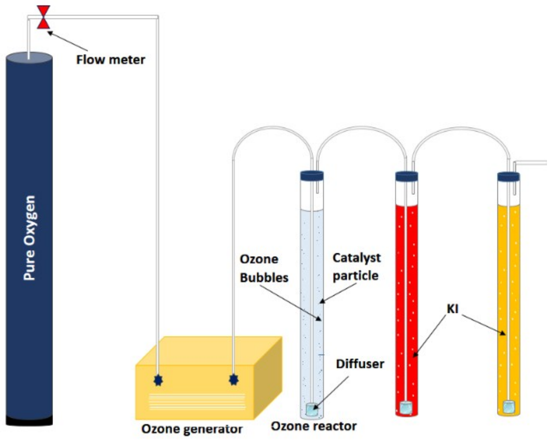

Fig. S1 –Catalytic ozonation process schematic diagram for pesticide destruction

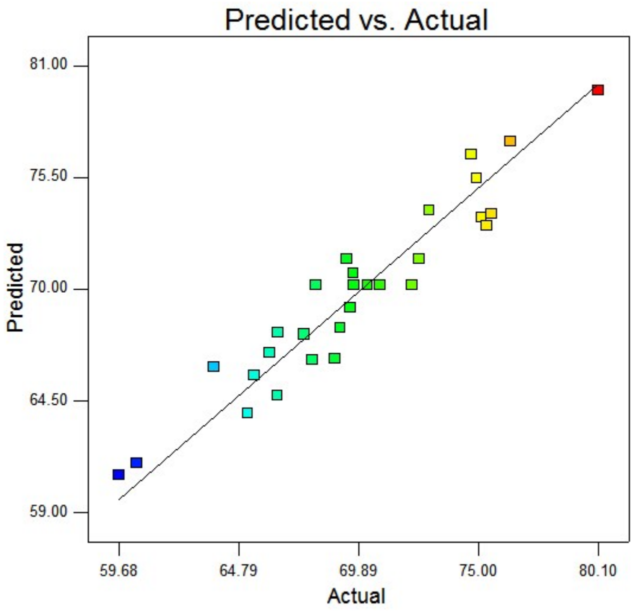

Fig. S2. Actual and predicted diazinon removal efficiency correlation

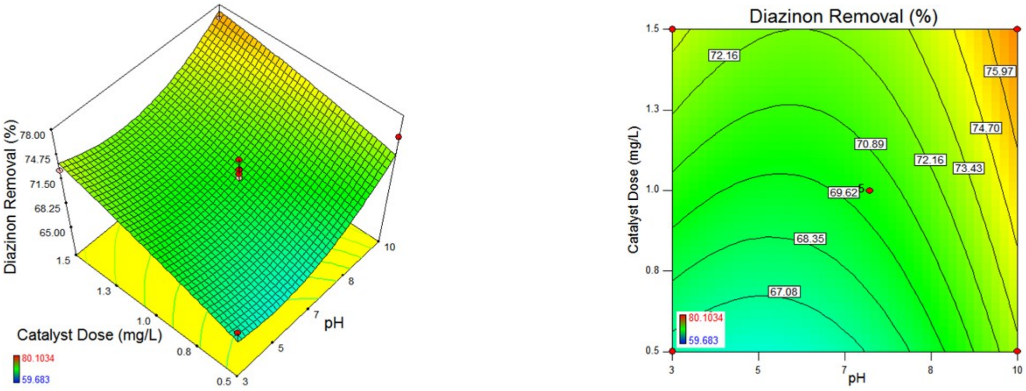

Fig. S3. The effect of pH and Catalyst loading on diazinon removal (time: 10 min and diazinon concentration: 10mg/L)

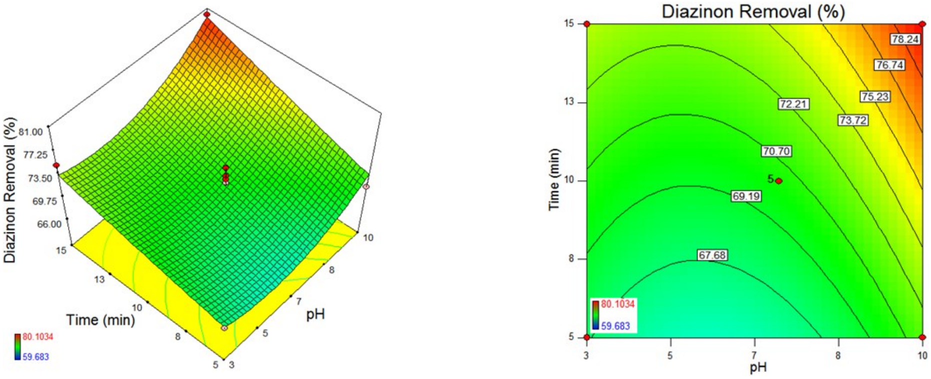

Fig. S4. The impact of pH and time on diazinon removal (catalyste loading: 1 mg/L and diazinon concentration: 10 mg/L)

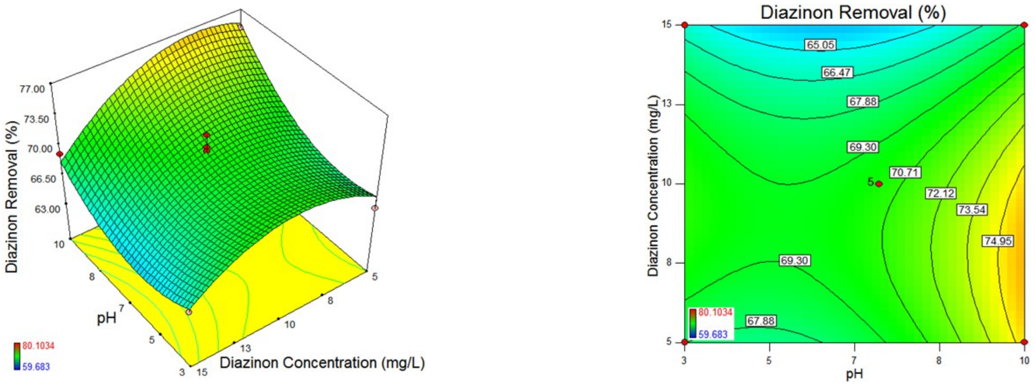

Fig. S5. The impact of pH and diazinon concentration on diazinon removal (catalyste loading: 1 mg/L and time: 10 min)

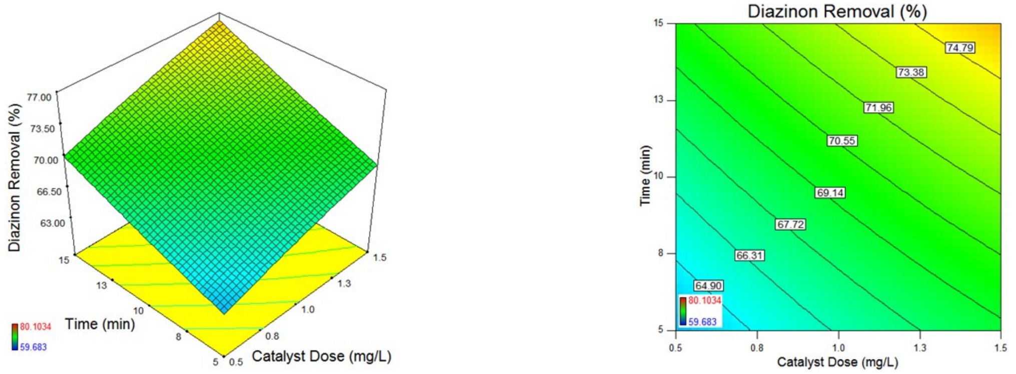

Fig.S6. The impact of catalyste loading and time on diazinon removal (pH: 7 and diazinon concentration: 10 mg/L)

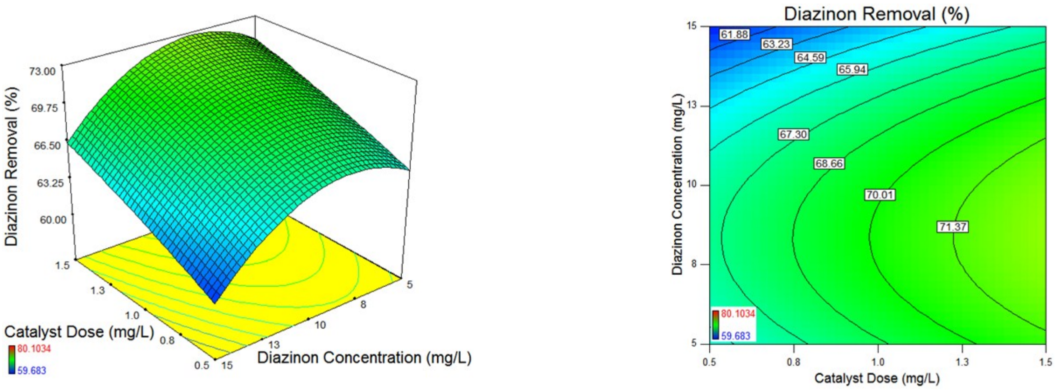

Fig. S7. The effect of catalyste loading and diazinon concentration time on diazinon removal (pH: 7 and time: 10 mg/L)

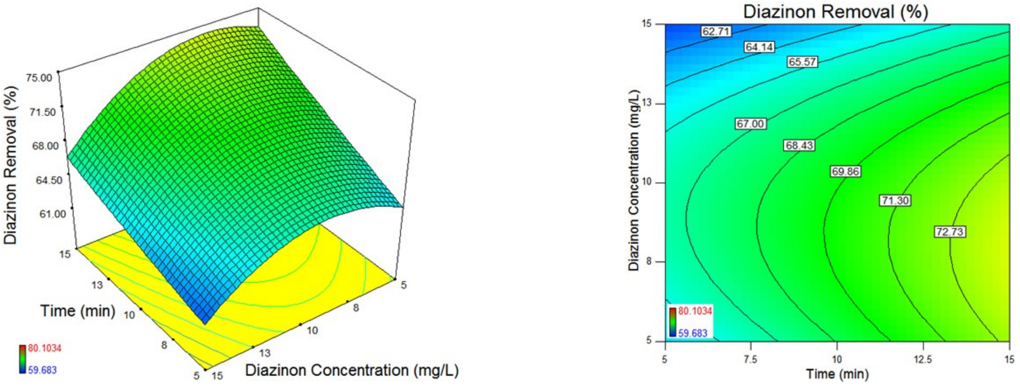

Fig. S8. The impact of time and diazinon concentration on diazinon removal (catalyste loading: 1mg/L and pH: 7)

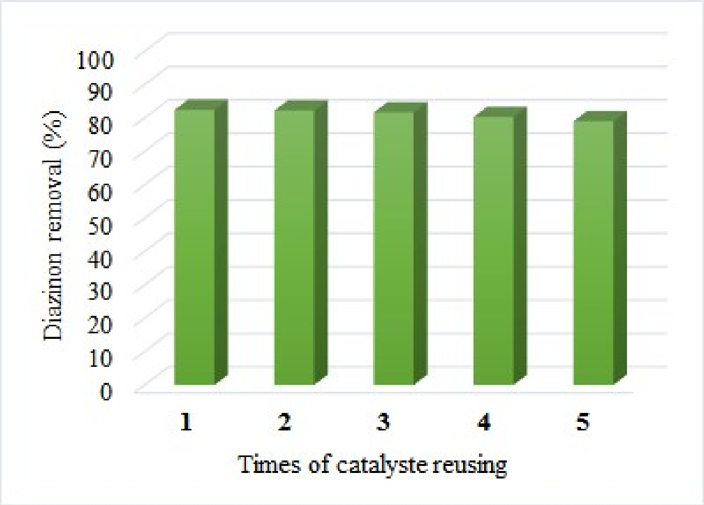

Fig. S9. Diazinon removal efficiency of Nano-MgO@CNT@Gr/O_3 throughout 5 runs
